# Indexing Resilience to Heat and Drought Stress in the Wild Relatives of Rapeseed-Mustard

**DOI:** 10.3390/life13030738

**Published:** 2023-03-09

**Authors:** Anamika Kashyap, Sujata Kumari, Pooja Garg, Ranjeet Kushwaha, Shikha Tripathi, Jyoti Sharma, Navin C. Gupta, Rajeev Ranjan Kumar, Rashmi Yadav, Harinder Vishwakarma, Jai Chand Rana, Ramcharan Bhattacharya, Mahesh Rao

**Affiliations:** 1 ICAR-National Institute for Plant Biotechnology, Delhi 110012, India; 2 Division of Forecasting and Agricultural System Modeling, ICAR-Indian Agricultural Statistics Research Institute, Delhi 110012, India; 3 ICAR-National Bureau of Plant Genetic Resources, Delhi 110012, India; 4 Bioversity International, India Office, Delhi 110012, India

**Keywords:** wild species, U triangle species, abiotic stress, climate change, stress tolerance

## Abstract

**Simple Summary:**

Wild species are weedy relatives and ancestors of domesticated crops that store economically important traits. Due to their natural tolerance to many biotic and abiotic stresses, they are widely used in plant breeding and crop improvement programs. Using a source of tolerance from crop wild relatives (CWRs), and introgressing the genetic factors into elite cultivars may improve resilience in modern crop cultivars. However, the lack of best practices and opportunities to systematically assess CWRs limits their use in crop improvement programs. The current study was conducted with Brassica’s wild and U-triangle species, which varied in their potential to withstand heat and drought stress, in an attempt to identify genotypes with a high degree of tolerance to abiotic stresses. Screening was performed at the germination and early seedling stages, for which morphological data and biochemical analyses were conducted.

**Abstract:**

Wild species are weedy relatives and progenitors of cultivated crops, usually maintained in their centres of origin. They are rich sources of diversity as they possess many agriculturally important traits. In this study, we analysed 25 wild species and 5 U triangle species of *Brassica* for their potential tolerance against heat and drought stress during germination and in order to examine the early seedling stage. We identified the germplasms based on the mean membership function value (MFV), which was calculated from the tolerance index of shoot length, root length, and biochemical analysis. The study revealed that *B. napus* (GSC-6) could withstand high temperatures and drought. Other genotypes that were tolerant to the impact of heat stress were *B. tournefortii* (RBT 2002), *D. gomez-campoi*, *B. tournefortii* (Rawa), *L. sativum*, and *B. carinata* (PC-6). *C. sativa* resisted drought but did not perform well when subjected to high temperatures. Tolerance to drought was observed in *B. fruticulosa* (Spain), *B. tournefortii* (RBT 2003), *C. bursa-pastoris* (late), *D. muralis*, *C. abyssinica* (EC694145), *C. abyssinica* (EC400058) and *B. juncea* (Pusa Jaikisan). This investigation contributes to germplasm characterization and the identification of the potential source of abiotic stress tolerance in the Brassica breeding programme. These identified genotypes can be potential sources for transferring the gene(s)/genomic regions that determine tolerance to the elite cultivars.

## 1. Introduction

The Brassicaceae family, comprising the Brassica genus, contains over 372 genera and 4060 species that are diverse and predominantly grown for edible, industrial oil, and vegetable purposes [1,2]. There are six important cultivated species, and their interspecific relations among the cultivated species can be understood by the ‘Triangle of U’ [3,4]. In the Indian context, *Brassica juncea* (L.) Czern, known as Indian mustard, with coverage of >95% of the total oilseed Brassica, is a significantly important oilseed crop. It is cultivated as a winter crop in tropical and subtropical climates (6 °C to 27 °C) under wide conditions, such as rainfed and irrigated, early and late-planted, and single or mixed-crop [5,6]. Brassica crops are vulnerable to continued climate change with increasing stresses due to abiotic factors, such as heat and drought, which negatively impact yields globally [7,8].

Under field conditions, plants are continuously exposed to environmental challenges due to high or low temperatures, drought, salinity, etc., and multiple stresses [9]. Consequently, the survival probability of the crops, growth-related parameters, physiological processes, and agricultural productivity are negatively affected by the field conditions [10]. In addition, the seed germination and seedling stages are more prone to cell injuries at high temperatures due to direct contact with the soil surface, as the soil temperature is generally 3–4 °C higher than the ambient temperature [11]. Similarly, drought exerts detrimental effects on plant physiology and metabolic processes, resulting in growth reduction and a lower accumulation of biomass [12]. Therefore, efforts to identify abiotic stress tolerant genotypes and their utilization in the crop breeding program are imperative for enhancing climatic resilience.

Crop wild relatives (CWRs) are widely utilized in plant breeding and crop improvement programs for their naturally tolerant behavior against many biotic and abiotic stresses [13,14]. One possible strategy for improving resilience in modern-day crop cultivars is to utilize an identified source of tolerance from the CWRs and introgress the genetic factors into the elite cultivars [15]. However, the lack of the best methodologies and opportunities to systematically assess the CWRs limits their utilization in crop improvement programs [16].

In a recent investigation, the thermotolerant genotypes were identified from the *Brassica juncea* at the seedling stage from 34 diverse genotypes [17]. Furthermore, a study on PEG-mediated drought stress on *Brassica rapa* L. cv. BARI Sharisha-15 resulted in the reduced growth of seedlings. However, a pre-treatment with exogenous osmolytes improved the growth, biomass accumulation, and other oxidative stress indicators related to antioxidant enzymatic activity [18]. Therefore, to identify genotypes with a high degree of tolerance to abiotic stresses, the present study was undertaken with Brassica’s wild and U triangle species, which varied in their potential to withstand heat and drought stress. Furthermore, morphological data analysis and biochemical assays were carried out at the germination and early seedling stages in order to index the tolerance across the genotypes.

## 2. Materials and Methods

### 2.1. Experimental Material

Thirty genotypes of the Brassica species, including 25 wild species and five Brassica spp. of the U triangle, were used in this study (Table 1). These wild species were collected from different sources previously and were maintained at the ICAR-National Institute for Plant Biotechnology, New Delhi, India. The mature seeds of each genotype were cleaned and surface-sterilized with sodium hypochlorite 1% (*w/v*) for 10 min, rinsed twice with distilled water and were used further for different treatments.

### 2.2. Stress Treatments

#### 2.2.1. Seed Germination Treatment

- Heat stress: Thirty seeds of each genotype were placed in Petri dishes (9 cm diameter) with a germinating sheet that was saturated with distilled water. The petri dishes were kept in a growth chamber and the heat treatment was given, with modifications to the previously described protocol [17]. In a light period of the 16/8 h light/dark cycle, seeds were exposed to a gradual elevation of temperature from 25 °C to 42 °C (Relative humidity ~ 45–50%). After the exposure of heat for 4 h at 42 °C, the temperature was gradually decreased to 25 °C (Relative humidity 70%) and the complete cycle was repeated for 5 days. Along with the treatment, a control experiment was carried out with optimum temperature 25 °C ± 2 °C (Relative humidity 70%) with a 16/8 h light/dark cycle.
- PEG-mediated drought stress: Seed germination was conducted with the same method as a previously described protocol, with modifications [19]. Thirty seeds of each genotype were placed in Petri dishes (9 cm diameter) with a germinating sheet that was saturated with three different concentrations (*w/v*) of PEG6000 (Thomas Baker, India), i.e., 2.5%, 5%, 10% and distilled water (control). All the Petri dishes were kept in a growth chamber set at 25 °C ± 2 °C initially in the dark for 2 days and subsequently kept under a 16/8 h light/dark cycle for the next 3 days of the treatment. Days to germination, germination percentage and data for root length and shoot length were recorded for the analysis in a replicated manner.

**Table 1:** List of crop wild relatives used in the present study.

S. No.	Genotypes	Chr No. (n) [20]	Native	S. No.	Species/Genotypes	Chr No. (n) [20]	Native
**1**	*Biscutella didyma*	-	Distributed over Mediterranean basin, central Europe, and southwest Asia [21]	**18**	*Lepidium sativum*	*-*	Temperate and sub-tropical regions [22]
**2**	* Brassica fruticulosa *	8	Europe [20]	**19**	*Sinapis alba*	12	Native of the Mediterranean and the Near East [23]
**3**	* Brassica fruticulosa * (Spain)	8		**20**	*Crambe abyssinica* (EC400058)	45	Distributed mainly in the Mediterranean, Euro-Siberian regions and northeast Africa [24]
**4**	*Brassica tournefortii* (RBT2002)	10	Native to arid and semi-arid regions of nothern Africa, Mediterranean areas of southern Europe and Middle-East [25]	**21**	*Crambe abyssinica* (EC694145)	45	
**5**	*Brassica tournefortii* (RBT2003)	10		**22**	*Eruca sativa* (IC57706)	11	Distributed in Europe and Western Asia. Originated from the Mediterranean region [26]
**6**	*Camelina sativa*	20	Originated from SE Europe and southwest Asia [27]	**23**	*Eruca sativa* (IC62713)	11	
**7**	*Capsella bursa-pastoris* (early)	16	Africa, Temperate and Tropical Asia and Europe [28]	**24**	Oxycamp (Synthetic amphidiploid of *B. oxyrrhina* and *B. rapa*)	-	Resynthesised at ICAR-NIPB, New Delhi
**8**	*Capsella bursa-pastoris* (late)	16					
**9**	*Diplotaxis assurgens*	9	Distributed in central Europe and Mediterranean region, mostly in nothernwest Africa [29]	**25**	*Brassica tournefortii* (Rawa)	10	Native to arid and semi-arid regions of nothern Africa, Mediterranean areas of southern Europe and Middle-East [25]
**10**	* Diplotaxis catholica *	9		**U Triangle species**			
**11**	*Diplotaxis cretacia*	-					
**12**	* Diplotaxis erucoides *	7		**26**	*Brassica rapa var. yellow sarson* (IC374272)	10	Mediterranean center with a secondary center in the Near East [30]
**13**	*Diplotaxis gomez-campoi*	-					
**14**	* Diplotaxis muralis *	21		**27**	*B. juncea* (Pusa Jaikisan)	18	Asiatic origin with its center of major diversity in China [23]
**15**	* Diplotaxis tenuisilique *	9		**28**	*B. nigra* (EC472708)	8	Widespread in central and southern Europe. Belongs to a Mediterranean center with a secondary center in the Near East [23]
**16**	*Diplotaxis viminea*	10		**29**	*B. napus*(GSC 6)	19	Formed on the coast of northern Europe, Mediterranean region [30]
**17**	* Enarthrocarpus lyratus *	10	-	**30**	*B. carinata* (PC-6)	17	Restricted to Ethiopia and neighboring territories [23]

#### 2.2.2. Seedling Treatments

- Heat stress: The seeds were sown in pots (6.5 cm in height and 7.5 cm in diameter) filled with homogenized field soil and allowed to germinate at the optimum temperature of 25 °C ± 2 °C under a 16/8 h light/dark cycle for 10 days in the plant growth chamber. Ten-day-old seedlings were exposed to heat treatment for 5 days, as described above and in previous protocol [17]. Data were recorded before and after the treatments, and the samples for biochemical analysis were collected in a replicated manner.
- PEG-mediated drought stress: The seeds were rolled in the germinating sheets, sized according to the depth of the germinating tray and were allowed to germinate for 10 days in a growth chamber set at an optimum temperature of 25 °C ± 2 °C under a 16/8 h light/dark cycle with a continuous distilled water supply from the bottom of the tray. A 5-day treatment was given to seedlings by replacing the distilled water of each tray with the PEG6000 (Thomas Baker, India) solution so that each tray had a different concentration (*w/v*) of PEG, i.e., 2.5%, 5% and 10%. A control experiment was maintained further for the next 5 days at the optimum temperature and supplied with distilled water. Data were recorded in a replicated manner, and the samples for the biochemical analysis were collected.

### 2.3. Germination and Survival Rate

The germination percentage was calculated using the data of the total number of seeds germinated and the number of seeds placed. The survival rate was determined by dividing the number of seedlings that survived by the total number of seedlings present at 5 days after treatment. These two parameters were analyzed using the following formula:

$$Germination\;percentage\;\left(GP\right)=\frac{no.\;of\;seeds\;germinated}{total\;number\;of\;seeds}\times 100$$

$$\;\;\;\;\;\;\;\;Survival\;Percentage\;\left(SP\right)=\frac{no.\;of\;seedlings\;survived}{total\;number\;of\;seedlings}\times 100$$

### 2.4. Biochemical Assays

#### 2.4.1. Lipid Peroxidation: Malondialdehyde (MDA) Content Analysis

Malondialdehyde (MDA) content was measured according to the previously described approach with minor adjustments [31]. For this, 0.5 g of tissue was homogenized in 5 mL of 0.1% trichloroacetic acid (TCA), followed by centrifugation for 5 min at 10,000 rpm. The supernatant was collected in which 4 mL of 20% TCA containing 0.5% thiobarbituric acid (TBA) was added to every 1 mL aliquot. The mixture was then heated for 30 min at 65 °C, followed by chilling in an ice bath. At 532 and 600 nm, the absorbance of the supernatant was recorded using a spectrophotometer (Evolution 300 UV-VIS, Thermoscientific, England, UK). The absorbance at 600 nm was subtracted from the absorbance at 532 nm to adjust unrelated turbidity. Finally, using an extinction coefficient of 155 mM^−1^ cm^−1^, the lipid peroxidation level was reported as µM of MDA produced.

#### 2.4.2. Proline Content

Proline content was measured according to the previously described protocol [32]. For this, 0.5 g of the frozen plant materials was homogenized in 10 mL of 3% sulphosalicylic acid, followed by filtration. Two mL of the extract was added with 2 mL acid ninhydrin and 2 mL glacial acetic acid in a test tube and incubated for 1 h at 100 °C, followed by a halt in the ice bath. The chromophore was separated by adding 4 mL of toluene with aggressive mixing for 15–20 s and was kept until the two phases separated. The toluene-containing chromophore was then transferred to another test tube, and the absorbance was measured at 520 nm using a spectrophotometer (Evolution 300 UV-VIS, Thermoscientific, England, UK).

### 2.5. Estimation of Tolerance Index (TI) and Membership Function Value (MFV)

The tolerance index (TI) was calculated based on shoot length (SL), root length (RL) and the biochemical parameters of the genotypes under controlled and treatment conditions according to [17,33]. The following equation was used for the calculation:

$$\mathrm{TI}ij=\frac{X_{s}^{ij}}{X_{ns}^{ij}}$$

where TI*ij* is the tolerant index of the trait (*j*) for the genotype (*i*), and $X_{s}^{ij}$ and $X_{ns}^{ij}$ are the values of the trait (*j*) for the genotypes (*i*) obtained under stressed (*s*) and non-stressed conditions (*ns*), respectively.

The stress tolerance index was derived by calculating the membership function value (MFV) using the following equations [33,34].

If a trait is positively correlated with tolerance, then

$$Uij=\frac{{\mathrm{TI}}_{\;ij}-{\mathrm{TI}}_{j\;min}}{{\mathrm{TI}}_{j\;max}-{\mathrm{TI}}_{j\;min}}$$

If a trait is negatively correlated with tolerance, then

$$Uij=1-\frac{{\mathrm{TI}}_{\;ij}-{\mathrm{TI}}_{j\;min}}{{\mathrm{TI}}_{j\;max}-{\mathrm{TI}}_{j\;min}}$$

where U*ij* is the MFV of the trait (*j*) for genotype and (*i*) for tolerance; ${\mathrm{TI}}_{j\;min}$ and ${\mathrm{TI}}_{j\;max}$ are the minimum and maximum values, respectively, for the tolerance index (TI*ij*) for the trait, and (*j*) is the tolerance index for genotype (*i*). The mean value of the MFV obtained from different traits was calculated, and the genotype’s tolerance was determined according to the average mean MFV values [33].

### 2.6. Statistical Analysis

All the statistical and Principal component analysis (PCA) were carried out using the SAS software package. Data collected were subjected to analysis of variance (ANOVA), and the means were compared through t-tests (LSD), and *p* ≤ 0.05 were considered significant. The correlation study was performed using the Pearson correlation method. Three independent biological replicates and results were displayed as mean ± standard deviation (SD) for physio-biochemical and molecular analysis.

## 3. Results

### 3.1. Stress Resilience of the Genotypes in Seed Germination and Survivability

**Figure 1:**
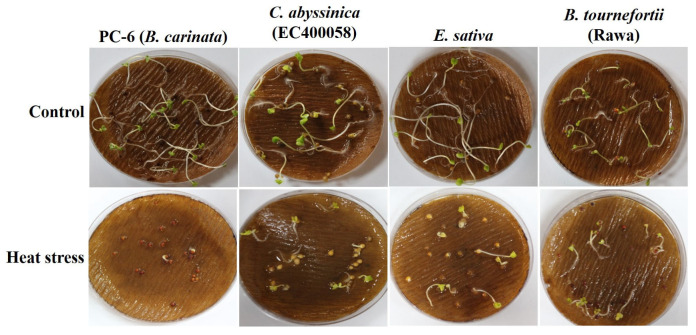
Seed germination of Brassica species grown under heat stress.

Seed germination was adversely impacted by the heat or the varied level of drought stress in all the genotypes (Table S1). Heat treatment affected seed germination, resulting in either an incline or decline in the average germination percentage (GP) under heat stress across the genotypes at varied levels (Figure 1). No significant effect of heat stress on GP was observed in *B. rapa* (IC374272), *Sinapis alba*, *L. sativum* and *C. sativa,* as they showed 100% germination in the control and treatments. The maximum increase in the germination percentage was observed in *B. tournefortii* cv. Rawa (38.46%), *C. sativa* (25%) and *D. assurgens* (12.5%) when exposed to heat treatment. The germination was affected the most in *B. fruticulosa* and *C. bursa-pastoris* (early), which were reduced by 67% and 63%, respectively. In our experiments, *E. lyratus* failed to germinate in the treatment, as well as in the control, while *D. erucoides* and *Oxycamp* could not attain the proper growth after germination under heat stress (Table S1). However, under stress treatment, the days to germination did not vary significantly except for in two genotypes; viz., *Oxycamp* and *B. didyma,* which showed delayed germination by two days under heat stress compared to their germination without stress.

**Figure 2:**
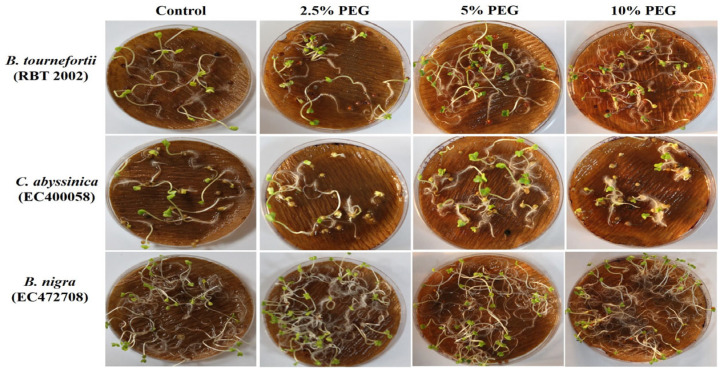
Seed germination of Brassica species grown under different variables of PEG.

In addition, no difference in the days to germination was observed under PEG treatments for any of the genotypes (Table S1). In PEG-mediated drought stress, the greatest change in GP was noted in *B. juncea* (Pusa Jaikisan), with a rise of 62.5%, 50% and 125% at PEG levels of 2.5%, at 5% and 10%, respectively. In contrast, in *B. fruticulosa,* the GP declined by 55%, 40% and 25% at 2.5%, 5% and 10% PEG, respectively. In *C. sativa*, *C. bursa-pastoris* (early and late), *B. nigra* (EC472708), and *B. carinata* (PC-6), no significant change in GP was noted due to the PEG treatments (Figure 2) (Table S1).

**Figure 3:**
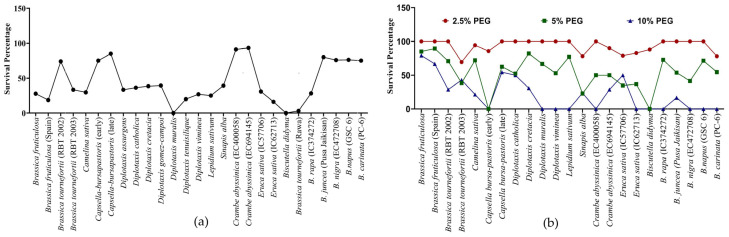
Survival percentage of Brassica seedlings after 5 days of (**a**) heat stress 42 °C and (**b**) PEG-mediated drought stress.

**Figure 4:**
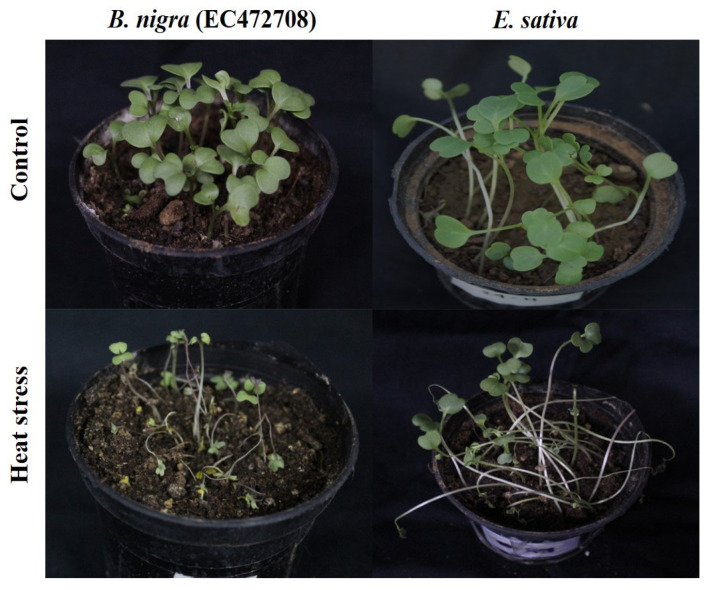
Effect of heat stress at 42 °C on Brassica seedlings.

Survivability reflects the genetic, physiological, biochemical and morphological capacity of the genotypes to cope with stress. For example, under heat stress, *D. muralis* and *Biscutella didyma* failed to survive after the heat treatment due to the intense impact of high temperature. In other genotypes, a decrease in the survival percentage (SP) was observed, which varied from as low as 3% in *B. tournefortii* (Rawa) to as high as 93% in *C. abyssinica* (EC694145) (Figure 3a and Figure 4).

In PEG-mediated drought stress, at 2.5% PEG for 5 days, seedlings of most of the genotypes remained unaffected in terms of survivability. Among the few affected genotypes, the lowest survivability of 69.4% was recorded in *Brassica tournefortii* (RBT 2003). Increasing the PEG concentration to 5% for 5 days greatly affected the survival of the seedlings. At 5% PEG, *Capsella bursa-pastoris* (early) and *Biscutella didyma* failed to survive, while the lowest survivability of 22.73% was observed in *Sinapis alba*. Further, when the concentration of PEG was increased to 10% for 5 days, some of the genotypes, namely, *Diplotaxis muralis*, *Diplotaxis viminea*, *Lepidium sativum*, *Eruca sativa* (IC62713), *Brassica rapa* (IC374272), *B. nigra* (EC472708), *B. napus* (GSC 6), and *B. carinata* (PC-6) did not survive (Figure 3b). The above results indicated a variable level of sensitivity in the genotypes to heat and drought stress at the germination and seedling stage, and also indexed the genotypes based on their resilience to heat and drought stress.

### 3.2. Effect of the Stresses on Shoot and Root Length

**Figure 5:**
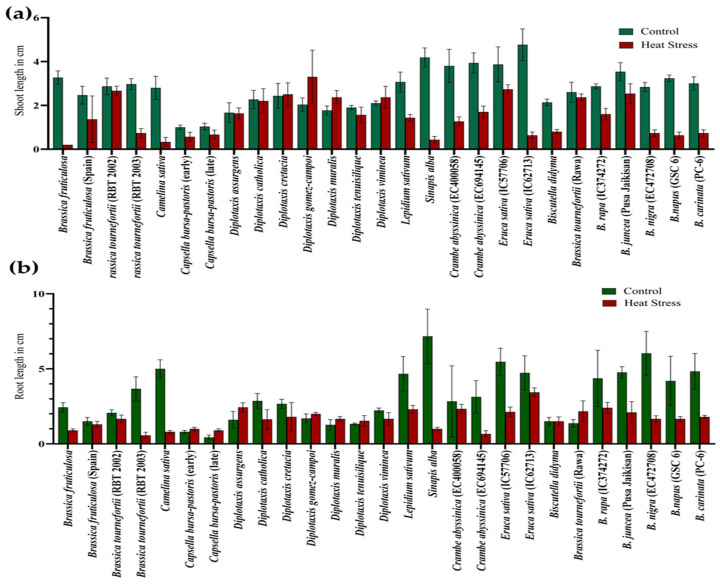
Effect of high temperature stress in *Brassica* spp. on shoot length (**a**) and root length (**b**).

During germination, exposure to the 5-day heat stress regime of 42 °C for 4 h in light on *Brassica* seeds negatively affected the shoot length (SL) and root length (RL) across the genotypes. The maximum reduction in shoot length was recorded in *Eruca sativa* (IC62713) and *Sinapis alba*. At the same time, the highest decrease in root length was evident in *S. alba*, followed by *B. nigra.* However, *D. gomez-campoi* showed a significant increase in the shoot length under heat-stress conditions, predicting a higher level of resilience to heat stress than the growth in unstressed conditions (Figure 5a,b) (Table S2).

**Figure 6:**
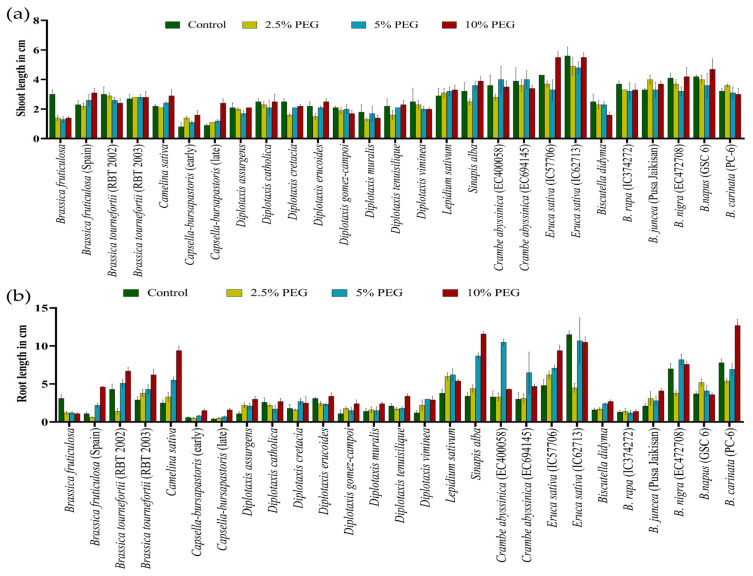
Effect of different concentrations of PEG-mediated drought stress in *Brassica* spp. on shoot length (**a**) and root length (**b**).

Similarly, under PEG-mediated variable drought stress, a significant variation in SL and RL at the germination stage was observed compared to controlled conditions (Figure 6a,b) (Table S3). The highest decrease in SL and RL was observed at all levels in *Brassica fruticulosa*. The performance of individual genotypes varied greatly under different treatments of PEG-mediated drought stress. Root length was noted to be significantly increased in the PEG treatments, which was its highest at 10% PEG, followed by 5% PEG and at its lowest at 2.5% PEG (Table S3).

### 3.3. Biochemical Assessment of Stress Response under Heat and Drought Stress at Early Seedling Stage: Lipid Peroxidation (MDA) and Proline Assays

**Figure 7:**
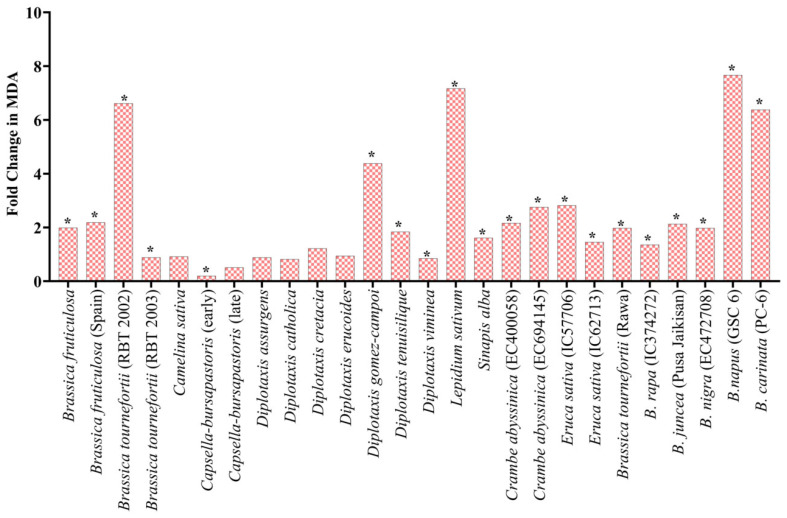
Fold change in MDA in seedling under heat stress w.r.t control. * Represents significance at the 0.05 level.

Under heat stress, the significant increase in the fold change (FC) of lipid peroxidation in the treated seedling ranged from highs of a 7.7-fold increase in *B. napus* (GSC 6) to lows of a 0.9-fold increase in *Brassica fruticulosa* (RBT 2003) at the juvenile stage. A significant fold change was observed in *L. sativum* (7.2 FC), *B. didyma* (7.1 FC), *B. tournefortii* (RBT 2002) (6.6 FC), *D. gomez-campoi* (4.4 FC), *B. carinata* (6.4 FC) and *D. muralis* (3.8 FC) (Figure 7). Under PEG-mediated drought stress conditions, the average increase in the proline content of the treated seedlings was significantly higher than their proline content under normal control conditions. For example, at 2.5% PEG, the highest 9.4-fold increase was observed in *D. muralis,* while only a 1.1-fold increase was observed in *B. tournefortii* (RBT 2003), *D. assurgens*, *D. viminea,* and *C. abyssinica* (EC694145) (Table 2). No change was noted in *S. alba* at 2.5% PEG; meanwhile, at 5% PEG, the highest proline level was recorded in *Crambe abyssinica* (EC694145) with a 17.2-fold increase, and the lowest proline level was recorded in *D. viminea* with a 1.1-fold change. Moreover, at 10% PEG treatment, *B. tournefortii* (Rawa) showed the highest 10.8-fold change, and *B. juncea* (Pusa Jaikisan) had the lowest 0.9-fold change (Table 2).

### 3.4. Identification of Tolerant Genotypes Based on Correlation, PCA, Tolerance Index (TI) and Membership Function Value (MFV)

The Pearson correlation coefficient ® was calculated to demonstrate the association among various observed traits in the individual experiments. In heat stress, no strong correlation between the morphological traits studied and between the survival percentage and MDA content was observed (Table 3 and Table 4). On the other hand, in drought stress, the various traits observed were found to have a significant positive association with each other; this is shown in Table 5 and Table 6. A significant positive correlation was seen between the RL and SL with different concentrations of PEG, while no effect of germination on RL, SL and SP on proline was observed.

**Figure 8:**
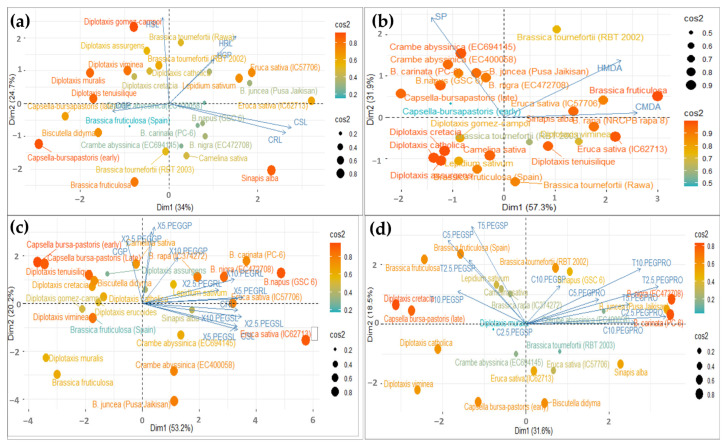
The Biplot showing Principal Component Analysis (PCA) to examine the importance of various observed traits contributing to heat stress tolerance at the germination stage (**a**), seedling stage (**b**) and drought stress tolerance at the germination stage (**c**), seedling stage (**d**), along with the distribution of genotypes studied. The variables used here are Control (C), Shoot Length (SL), Root Length (RL), Heat (H), Germination Percentage (GP), Survival Percentage (SP), MDA (MDA), Proline (PRO) and Treatments (X and T at 2.5 PEG, 5 PEG and 10 PEG).

A principal component analysis (PCA), based on the various attributes studied in the individual experiments, was performed to detect potential heat and drought-tolerant genotypes. In heat stress, analysis was performed to identify potentially tolerant genotypes at the germination and seedling stage. The variables were grouped into two main components that accounted for 58.71% and 89.17% of the total variability in the germination and seedling dataset; these had an eigenvalue > 1 (Figure 8a,b).

The variables used here are Control Shoot Length (CSL), Control Root Length (CRL), Heat Shoot Length (HSL), Heat Root Length (HRL), Control Germination Percentage (CGP), and Heat Germination Percentage (HGP).

In Figure 8a, the biplot diagram shows that the first principal component (Dim1) accounted for the maximum variability in the dataset (i.e., 34%), and that it had a strong positive correlation with heat root length (HRL) and heat germination percentage (HGP). In contrast, PC1 is negatively correlated with the heat shoot length (HSL). Further, in Figure 8b, the biplot diagram shows that the first principal component (Dim 1) accounted for the maximum variability in the dataset (i.e., 57.3%), and that it had had a strong positive correlation with heat MDA (HMDA) and a strong negative correlation with survival percentage (SP). The results indicated that RL, GP and MDA could identify the tolerant genotype that works well under heat-stressed conditions.

In drought stress, the variables were grouped into two main components that accounted for 53.2% and 31.6% of the total variability in the germination and seedling dataset; these had an eigenvalue > 1 (Figure 8c,d). In Figure 8c, the biplot diagram shows that the first principal component (Dim1) accounted for the maximum variability in the dataset (i.e., 53.2%), and had a strong positive correlation with 2.5% PEG GP and RL, 5% PEG GP and RL, and 10% PEG GP and RL. In contrast, PC1 was negatively correlated with 2.5% PEG SL, 5% PEG SL, and 10% PEG SL heat. Further, in Figure 8d, the biplot diagram showed that the first principal component (Dim 1) accounted for maximum variability in the dataset (i.e., 31.6%), and had a strong positive correlation with 2.5% PEG, 5% PEG, and 10% PEG proline, and strong negative correlation with 2.5% PEG, 5% PEG, and 10% PEG survival percentage. The results indicated that germination percentage, root length and proline content could identify the tolerant genotype that performs well under drought conditions.

The membership function value (MFV) of heat and drought tolerance was used as a comprehensive index to evaluate potentially tolerant genotypes. The estimated MFV values of the genotypes, based on traits studied under stress conditions, are mentioned in Tables S2, S4–S9. In our study, the MFV was the cumulative outcome of the TI of all the traits studied; this includes GP, RL and SL for screening at the germination stage and SP and biochemical assay (MDA for heat stress and proline for PEG-mediated drought stress) for screening at the seedling stage.

According to Rai et al. [17], the higher the TI value, the higher the MFV value. After this, the average MFV(s) value was calculated, concluding which tolerant genotype was identified. Under heat stress at the germination stage, a maximum mean MFV of 1.0 was recorded in *D. gomez-campoi* and *B. tournefortii* (Rawa). At the same time, a lower value was observed as 0.1 in *C. sativa, Sinapis alba* and *C. abyssinca* (EC694145). At the seedling stage, the highest mean MFV of 3.9 was observed in *B. napus* (GSC 6) and the lowest of 0.27 was observed in *D. catholica* (Table S4). In PEG-mediated drought stress at the germination stage, the mean MFV ranged between −0.17 in *B. fruticulosa* and 1.39 in *D. assurgens*. In PEG-mediated drought stress at the seedling stage, the lowest mean MFV was recorded in *D. viminea* at 0.46 and the highest mean MFV was recorded in *D. muralis* at 4.48. Data for the TI, MFV and Mean MFV are represented in Table S7.

## 4. Discussion

Global agriculture has started facing the challenge of climate change, in which extreme abiotic stresses often affect the crops severely [35]. The genus Brassica represents a large group of oilseed and vegetable crops throughout the world. Breeding tolerance to heat and drought has been imperative for the sustainability of productivity in the face of the increasing demand for edible oil [4]. In the process of the domestication and continuous breeding of the crop for a higher yield, the cultivated plant types of Brassica spp. have lost the genetic variability that aids stress resilience. At the same time, the Brassicaceae family includes a large reservoir of crop wild relatives (CWRs), showing intrinsic tolerance to many of the abiotic stresses [36]. In the case of abiotic stress, many of the studies on Brassica target mostly the vegetative and flowering stages of the plant [8], and reports on its genotypic tolerance to heat and drought at the early stages of the crops’ plant development are obscure. Here, we set out to screen the 25 wild species and 5 U triangle species of Brassica to identify genotypes that can withstand heat and drought stress in germination and the early growth of the seedlings.

In plants, high temperature at the sowing time lowers seed germination, potentially triggering seedling mortality, leading to poor crop stand and low seed yield [37]. Prior studies have demonstrated that transient daily heat stress during flowering in canola (*Brassica napus* L.) poses an increasing threat to grain production in this oilseed crop [38]. In leafy vegetables, exposure to a temperature of 40 °C decreased seed germination [39]. This decrease in seed germination is due to cell death, which negatively impacts the seedling establishment rate in wheat [40]. However, in our study, the lack of change in the germination of *B. rapa* at high temperatures indicated that this genotype is adapted to high day temperatures; this is characteristic of crops that grow in tropical and subtropical regions [41]. Similarly, in our screening, high temperature was found to negatively affect seed germination across most genotypes. Interestingly, an increase in the germination percentage of *D. assurgens* (mean MFV 0.9), *B*. *tournefortii* (Rawa) (mean MFV 1.0) and *L. sativum* (mean MFV 0.5) under heat stress suggested that these genotypes had a higher to moderate tolerance for heat stress at the germination stage.

We observed that, due to the gradual increase in the daily temperature from 25 °C to 42 °C, GP and early seedling growth gradually decreased, suggesting a direct effect of the high temperature on the factors involved in germination and seedling establishment. Several other studies indicated that roots are more sensitive to heat stress than shoots [42]. In this study, root and shoot growth decreased with heat stress, but the inhibitory effect was more prominent in roots. A decrease in the seed germination rate and shoot length due to high temperatures was demonstrated in tomatoes by Singh et al. [43] and in barley and radish by Cavusoglu et al. [44]. However, the genotypes *D. gomez-campoi* (mean MFV 1.0) and *D. viminea* (mean MFV 0.6) showed a rise in the shoot length under heat stress, possibly suggesting their potential tolerance to heat.

The survival percentage after a 5-day cycle of heat stress reflects the germplasm’s recovery potential [17]. MDA is frequently used to estimate lipid peroxidation levels, an essential biochemical parameter during stress in crop plants. Rashid et al. [45] observed an increase in MDA ranging from 35 to 50% in heat-stressed seedlings. Under high temperature and humidity stress, soybean plants began to deteriorate at seed development and maturity due to the accumulation of reactive oxygen species (ROS) in developing seeds, which was correlated with a higher level of lipid peroxidation [46]. In our study, the survival percentage of the seedlings and MDA content showed a negative correlation across the germplasms. However, a significantly higher MDA content in stressed Brassica genotypes was observed, though survival percentage did not always correlate with the level of thiobarbituric acid reactive substances (TBARS) [17].

Drought stress causes major losses in plant growth, leading to considerable yield losses in many crops worldwide [47]. Simulating drought stress with a PEG hypertonic solution is effective for screening during seed germination and the development of 24 rapeseed genotypes [48]. A study by Batool et al. [48] found that all the cultivars they evaluated experienced negative effects from drought stress. However, the severity of these effects differed among the cultivars, suggesting varying levels of tolerance to drought. The present results showed that seed GP was enhanced under PEG treatments in most genotypes compared to the untreated lot. However, in some genotypes, a decline in GP was also observed. A reduction in seed germination percentage by drought stress may be attributed to the reduced infusibility of water through the seed coat and initial water uptake by the seeds of durum wheat and sesame cultivars under stress conditions [49,50].

Similarly, a deficit of seed hydration due to high osmotic potential causes the inhibition of mechanisms that lead to the radicle emerging from the coat; thus, seed germination gets delayed [51]. Some plants can adapt to drought stress, and low stress can enhance seed germination, whereas high stress inhibits growth [52]. We observed a wide variation in seed GP among the wild species, which is not uncommon and is considered an intrinsic ability of the genotype [53]. Saeidnejad et al. [54] reported that such differences in black cumin seed germination could be due to genetic variability among the seeds, depending on the latitude or natural habitat at which accessions of seeds were collected. We also observed an increase in the germination percentage of some of the genotypes under PEG with a maximum of 10% compared to the untreated lot, similar to the observation in soybean by Begum et al. [19]. The members of the U triangle species did not show any significant differences in germination percentage under different levels of PEG concentration. The shoot length showed a more variable response across the genotypes under heat and drought stress. While the decrease in shoot length with an increase in the concentration of PEG was the common response across the genotypes, the shoot length observed at 2.5% PEG treatment was much higher than the shoot length without PEG. A similar observation was recorded in various oilseed crops, including *Carthamus tinctorius*, *Sesamum indicum,* and *B. napus*; this is because, under drought stress, the shoot length of the genotypes decreased when grown under PEG treatments [55,56,57]. The root length at an early seedling stage was increased significantly at each PEG treatment than when compared to non-treated plants. Kage et al. [58] reported that, in cauliflower, more root length under drought was due to the increased partitioning of assimilates towards the roots at the expense of shoot growth. Under drought stress, the elongated roots can be beneficial to obtaining water from the deeper soil layer. Similarly, in previous studies, under drought stress, higher RL in rapeseed mustard genotypes was also observed [57,59].

Drought stress can have a negative impact on various aspects of rapeseed growth and development, including germination, seedling establishment and shoot elongation [60]. Therefore, the successful establishment of a plant population depends on the adaptive aspects of seed germination and early seedling growth [61]. Our study showed a decline in seedling survivability as the concentration of PEG treatment was increased beyond 2.5%. In response to external osmotic stress, plants accumulate a wide range of organic solutes that undergo impending changes to deal with environmental factors.

Proline is well-known for its osmotic adaptation activity and role in the enhancement of the stress response by protecting cellular membranes and enzyme integrity [62]. PEG-treated seedlings had a significantly higher proline content than control plants (non-treated). Higher levels of proline content in plants treated with 5% and 10% of PEG6000 were also observed in the leaves and roots of two cultivars of *B. napus* (GSC 6) [63]. Most wild species, such as *B. fruticulosa*, *B. fruticulosa* (spain), *B. tornefortii* (RBT 2002 and 2003), and *C*. *sativa* show a higher increase in proline content at 10% PEG treatment when compared to the control (non-treated). A decreased fold change in other genotypes at 10% PEG, such as *D. muralis*, *Crambe abyssinica* (EC694145) and *Eruca sativa* (IC57706 and IC62713), was observed. Furthermore, the U triangle species also showed a decline in the proline content at 10% PEG. Plant survival, stress tolerance, and biochemical changes under stressed conditions, such as drought and salinity, depend on non-structural carbohydrates [64]. Furthermore, the correlation study showed that RL and SL under drought stress were not strongly associated with the germination percentage. Similarly, no positive correlation between the survival percentage and proline content could be established, which was significant in certain genotypes.

We further analyzed the relationship between the MFV of the heat and drought tolerance and the TI of the studied parameter, in order to identify the tolerant genotypes. The membership function of a fuzzy set is a generalization of the indicator function in classical sets; it represents the degree of truth as an extension of valuation [65]. Under heat stress, the mean MFV of the selected traits was calculated, and the genotypes with MFVs higher than the average were scored as potentially tolerant. The average values calculated were 0.5, 1.1, 0.5, and 1.7 for heat stress at germination, heat stress at the seedlings, PEG-mediated drought stress at germination, and PEG-mediated drought stress at the seedling stage, respectively.

## 5. Conclusions

A wide range of genotypic variability was observed in Brassica crop wild relatives and U triangle species under heat and PEG-mediated drought stress at the germination and early seedling stages. The identification of tolerant genotypes using morphological parameters and enzyme assays was supported by statistical analyses, including correlation, PCA, and MFV. We found that the traits studied were reliable parameters for indexing the tolerance across the genotypes. The identified genotypes can be further subjected to pre-breeding work for generating genetic stocks tolerant to heat and drought stress in the cultivated species, and these can serve as potential parents for the development of climate resilience varieties.

## Figures and Tables

**Table 2:** Proline content of wild and U triangle species of Brassica germinated under different concentrations of PEG6000. Different letters denote a significant difference at *p* < 0.05 based on the least significant difference (LSD) test.

S.No.	Genotype	Control	PEG 2.50%	FC	Control	PEG 5%		Control	PEG 10%	FC *
1	*Brassica fruticulosa*	0.13 ± 0.008 ^N^	0.243 ± 0.001 ^O^	1.8	0.135 ± 0 ^EFG^	0.629 ± 0.001 ^J^	4.7	0.067 ± 0 ^S^	0.506 ± 0 ^M^	7.5
2	*Brassica fruticulosa (Spain)*	0.22 ± 0.012 ^K^	0.745 ± 0.001 ^G^	3.3	0.092 ± 0 ^IHFG^	0.138 ± 0.001 ^O^	1.5	0.077 ± 0 ^Q^	0.779 ± 0.001 ^I^	10.1
3	*Brassica tournefortii* (RBT 2002)	0.524 ± 0.012 ^E^	0.815 ± 0.001 ^E^	1.6	0.311 ± 0 ^CB^	0.702 ± 0.001 ^I^	2.3	0.104 ± 0 ^K^	1.073 ± 0.001 ^F^	10.3
4	*Brassica tournefortii* (RBT 2003)	0.515 ± 0.001 ^F^	0.576 ± 0.001 ^K^	1.1	0.116 ± 0 ^EHFG^	0.165 ± 0.001 ^N^	1.4	0.105 ± 0.001 ^K^	1.137 ± 0.001 ^D^	10.8
5	*Camelina sativa*	0.15 ± 0.001 ^M^	0.415 ± 0.002 ^M^	2.8	0.143 ± 0 ^EFG^	0.849 ± 0.002 ^H^	5.9	0.127 ± 0 ^J^	1.259 ± 0 ^B^	9.9
6	*Capsella bursa-pastoris (early)*	0.091 ± 0 ^O^	0.117 ± 0.002 ^P^	1.3	0.158 ± 0.001 ^EFG^	0.534 ± 0.001 ^K^	3.4	0.098 ± 0.001 ^L^	0.206 ± 0.001 ^U^	2.1
7	*Capsella bursa-pastoris (late)*	0.051 ± 0.001 ^Q^	0.099 ± 0.001 ^P^	1.9	0.123 ± 0 ^EHFG^	0.209 ± 0.001 ^M^	1.7	0.078 ± 0.001 ^Q^	0.508 ± 0.001 ^M^	6.5
8	*Diplotaxis catholica*	0.142 ± 0.001 ^M^	0.308 ± 0.001 ^N^	2.2	0.038 ± 0 ^I^	0.165 ± 0.001 ^N^	4.3	0.09 ± 0.001 ^N^	0.241 ± 0.002 ^S^	2.7
9	*Diplotaxis cretacia*	0.059 ± 0.001 ^QP^	0.106 ± 0.001 ^P^	1.8	0.091 ± 0 ^IHFG^	0.135 ± 0.002 ^O^	1.5	0.098 ± 0.001 ^L^	0.213 ± 0.001 ^T^	2.2
10	*Diplotaxis muralis*	0.063 ± 0.001 ^P^	0.593 ± 0.037 ^KJ^	9.4	0.166 ± 0.181 ^EF^	1.629 ± 0 ^A^	9.8	0.093 ± 0.001 ^M^	0.457 ± 0.001 ^O^	4.9
11	*Diplotaxis viminea*	0.098 ± 0.001 ^O^	0.111 ± 0.001 ^P^	1.1	0.058 ± 0 ^IH^	0.064 ± 0.001 ^P^	1.1	0.037 ± 0.001 ^T^	0.068 ± 0.001 ^V^	1.8
12	*Lepidium sativum*	0.175 ± 0.001 ^L^	0.414 ± 0.001 ^M^	2.4	0.265 ± 0.001 ^CB^	1.02 ± 0.001 ^F^	3.8	0.187 ± 0 ^G^	0.72 ± 0.001 ^K^	3.9
13	*Sinapis alba*	0.592 ± 0.001 ^C^	0.6 ± 0.001 ^J^	1.0	0.124 ± 0 ^EHFG^	0.628 ± 0.029 ^J^	5.1	0.813 ± 0 ^B^	0.961 ± 0.001 ^G^	1.2
14	*Crambe abyssinica*(EC400058)	0.276 ± 0.001 ^I^	0.693 ± 0.034 ^H^	2.5	0.767 ± 0.001 ^A^	1.642 ± 0.028 ^A^	2.1	0.072 ± 0 ^R^	0.723 ± 0.001 ^J^	10.0
15	*Crambe abyssinica* (EC694145)	0.236 ± 0.001 ^J^	0.254 ± 0.001 ^O^	1.1	0.082 ± 0.001 ^IHG^	1.46 ± 0.001 ^C^	17.8	0.337 ± 0.001 ^E^	0.396 ± 0 ^Q^	1.2
16	*Eruca sativa* (IC57706)	0.378 ± 0.002 ^H^	0.669 ± 0.001 ^I^	1.8	0.243 ± 0.001 ^CD^	1.105 ± 0 ^E^	4.5	0.166 ± 0.001 ^H^	0.312 ± 0.001 ^R^	1.9
17	*Eruca sativa* (IC62713)-3	0.277 ± 0.002 ^I^	0.523 ± 0.002 ^L^	1.9	0.129 ± 0.001 ^EHFG^	0.967 ± 0.002 ^G^	7.5	0.082 ± 0.001 ^P^	0.445 ± 0.002 ^P^	5.5
18	*Biscutella didyma*	0.439 ± 0.001 ^G^	0.588 ± 0.001 ^KJ^	1.3	0.107 ± 0 ^IEHFG^	0.135 ± 0.001 ^O^	1.3	0.086 ± 0.001 ^O^	0.481 ± 0.002 ^N^	5.6
19	*B. rapa* (IC374272)	0.229 ± 0.001 ^KJ^	0.774 ± 0.001 ^F^	3.4	0.179 ± 0.001 ^ED^	0.512 ± 0.001 ^L^	2.9	0.19 ± 0 ^F^	0.563 ± 0.001 ^L^	3.0
20	*B. juncea* (Pusa Jaikisan)	0.582 ± 0.001 ^D^	0.864 ± 0.001 ^D^	1.5	0.119 ± 0 ^EHFG^	1.582 ± 0.019 ^B^	13.3	1.241 ± 0 ^A^	1.078 ± 0.001 ^E^	0.9
21	*B. nigra* (EC472708)	0.665 ± 0.001 ^A^	1.018 ± 0.001 ^B^	1.5	0.329 ± 0 ^B^	1.573 ± 0.001 ^B^	4.8	0.465 ± 0 ^D^	1.382 ± 0.001 ^A^	3.0
22	*B. napus* (GSC 6)	0.612 ± 0.001 ^B^	0.89 ± 0.001 ^C^	1.5	0.138 ± 0 ^EFG^	1.097 ± 0.001 ^E^	8.0	0.147 ± 0.001 ^I^	0.958 ± 0.001 ^H^	6.5
23	*B. carinata* (PC-6)	0.588 ± 0.001 ^DC^	1.049 ± 0.001 ^A^	1.8	0.299 ± 0 ^CB^	1.305 ± 0.001 ^D^	4.4	0.606 ± 0 ^C^	1.231 ± 0.001 ^C^	2.0

* FC: Fold change.

**Table 3:** Correlation between traits studied under heat stress at the germination stage. Values represent Pearson’s correlation coefficient with * significance at the 0.05 level (1-tailed) and ** significance at the 0.01 level (1-tailed).

Traits	CGP	HGP	CRL	HRL	CSL	HSL
CGP	1					
HGP	0.158	1				
CRL	−0.076	0.332 *	1			
HRL	−0.166	0.274	0.143	1		
CSL	−0.380 *	−0.001	0.742 **	0.274	1	
HSL	−0.123	0.158	−0.246	0.350 *	−0.127	1

**Table 4:** Correlation between traits studied under heat stress at the seedling stage. Values represent Pearson’s correlation coefficient with * significance at the 0.05 level (1-tailed) and ** significance at the 0.01 level (1-tailed).

Traits	SP	CMDA	HMDA
SP	1		
CMDA	−0.347 *	1	
HMDA	−0.051	0.604 **	1

The variables used here are Survival Percentage (SP), Control MDA (CMDA), and Heat MDA (HMDA).

**Table 5:** Correlation between traits studied under PEG-mediated drought stress at germination stage. Values represent Pearson’s correlation coefficient with * significance at the 0.05 level (1-tailed) and ** significance at the 0.01 level (1-tailed).

		CSL	CRL	2.5% PEG		5% PEG		10% PEG		CGP	2.5% PEG GP	5% PEG GP	10% PEG GP
				SL	RL	SL	RL	SL	RL				
Control	SL	**1**											
	RL	0.821 **	1										
2.5% PEG	SL	0.894 **	0.814 **	1									
	RL	0.516 **	0.597 **	0.622 **	1								
5% PEG	SL	0.876 **	0.696 **	0.879 **	0.491 **	1							
	RL	0.793 **	0.874 **	0.821 **	0.673 **	0.836 **	1						
10% PEG	SL	0.826 **	0.728 **	0.839 **	0.553 **	0.844 **	0.779 **	1					
	RL	0.646 **	0.825 **	0.695 **	0.662 **	0.661 **	0.916 **	0.731 **	1				
CGP		−0.267	0.023	−0.314	−0.017	−0.377 *	0.014	−0.225	0.204	1			
2.5% PEG GP		−0.178	0.091	0.016	0.119	−0.074	0.11	0.074	0.284	0.397 *	1		
5% PEG GP		−0.113	0.077	0.022	0.131	−0.038	0.089	0.093	0.219	0.364 *	0.691 **	1	
10% PEG GP		0.164	0.268	0.261	0.332 *	0.177	0.285	0.234	0.256	0.096	0.209	0.573 **	1

The variables used here are Control, Shoot Length (SL), Root Length (RL), Shoot Length (SL), Heat Root Length (RL), Germination Percentage (GP).

**Table 6:** Correlation between traits studied under PEG-mediated drought stress at the seedling stage. Values represent Pearson’s correlation coefficient with * significance at the 0.05 level (1-tailed) and ** significance at the 0.01 level (1-tailed).

		2.5% PEG SP		5% PEG SP		10% PEG SP		2.5% PEG PRO		5% PEG PRO		10% PEG PRO	
		C	T	C	T	C	T	C	T	C	T	C	T
2.5% PEG SP	C	1											
	T	0.342	1										
5% PEG SP	C	0.079	0.432 *	1									
	T	−0.007	0.612 **	0.740 **	1								
10% PEG SP	C	−0.09	−0.184	0.165	0.02	1							
	T	0.186	0.005	0.292	0.34	0.441 *	1						
2.5% PEG PRO	C	−0.291	−0.366 *	−0.337	−0.276	0.301	−0.219	1					
	T	−0.373 *	−0.106	−0.165	0.025	0.195	−0.293	0.808 **	1				
5% PEG PRO	C	−0.168	0.092	−0.159	−0.01	0.128	−0.309	0.209	0.405 *	1			
	T	−0.201	0.056	−0.265	0.017	−0.064	−0.406 *	0.343	0.510 **	0.540 **	1		
10% PEG PRO	C	−0.278	−0.154	−0.379 *	−0.155	0.22	−0.154	0.592 **	0.443 *	−0.02	0.441 *	1	
	T	−0.31	−0.137	0.09	0.129	0.295	−0.113	0.724 **	0.722 **	0.298	0.379 *	0.475 *	1

The variables used here are Control, ©, Treatment (T), Survival Percentage (SP), Proline (PRO).

## Data Availability

The data that supports the findings of this study are available in the supplementary material of this article.

## Supplementary Materials

The following supporting information can be downloaded at: https://www.mdpi.com/article/10.3390/life13030738/s1, Table S1: Percent decrease in germination for different *Brassica* sp. under stress conditions over control; Table S2: Tolerance index (TI) and membership function value (MFV) for trait germination percentage, shoot length and root length under heat stress; Table S3: Shoot length and root length of wild and U triangle species of Brassica germinated under different concentrations of PEG6000. Different letters denote a significant difference at *p* < 0.05 based on the least significant difference (LSD) test; Table S4: Tolerance index (TI) and membership function value (MFV) for trait survival percentage and MDA under heat stress; Table S5: Tolerance index (TI) and membership function value (MFV) for trait shoot length and root length at 2.5% PEG6000; Table S6: Tolerance index (TI) and membership function value (MFV) for trait shoot length and root length at 5% and 10% PEG6000; Table S7: Tolerance index (TI) and membership function value (MFV) for trait germination percentage at 2.5%, 5% and 10% PEG6000; Table S8: Tolerance index (TI) and membership function value (MFV) for trait survival percentage at 2.5%, 5% and 10% PEG6000; Table S9: Tolerance index (TI) and membership function value (MFV) for trait proline content at 2.5%, 5% and 10% PEG6000.
