# Supplementary material for: Indexing Resilience to Heat and Drought Stress in the Wild Relatives of Rapeseed-Mustard
Source: Life (Basel). 2023 Mar 9;13(3):738. doi: 10.3390/life13030738 (PMC10055847; doi:10.3390/life13030738)
Supplement: Supplementary file 1 [file life-13-00738-s001.zip › life-2177374-supplementary.pdf]

Table S1- Percent decrease in germination for different *Brassica* sp. under stress conditions over control

S.no.	Species /Genotypes	Days to Germination						Germination Percentage									
		Heat		Drought				Heat			Drought						
		C	T	C	2.5% PEG	5% PEG	10% PEG	C	T	%Change	C	2.5% PEG	%Change	5% PEG	%Change	10% PEG	%Change
**1**	*Brassica fruticulosa*	1	1	1	1	1	1	90	30	66.67 ↓	100	45	55↓	60	40↓	75	25↓
**2**	*Brassica fruticulosa (Spain)*	3	3	3	3	3	3	65	30	53.85↓	80	100	25↑	100	25↑	50	37.5↓
**3**	*Brassica tournefortii (RBT 2002)*	1	1	1	1	1	1	85	75	11.76↓	90	95	5.55↑	95	5.55↑	100	11.11↑
**4**	*Brassica tournefortii (RBT 2003)*	1	1	1	1	1	1	85	80	5.88↓	90	90	0	100	11.11↑	100	11.11↑
**5**	*Camelina sativa*	1	1	1	1	1	1	90	90	0	100	100	0	100	0	100	0
**6**	*Capsella bursa-pastoris (early)*	3	3	3	3	3	3	95	35	63.16↓	100	100	0	100	0	100	0
**7**	*Capsella bursa-pastoris (late)*	3	3	3	3	3	3	85	80	5.88↓	100	100	0	100	0	100	0
**8**	*Diplotaxis assurgens*	1	1	1	1	1	1	80	90	12.50↑	90	95	5.55↑	100	11.11↑	100	11.11↑
**9**	*Diplotaxis catholica*	1	1	1	1	1	1	95	90	5.26↓	80	95	18.75↑	95	18.75↑	100	25↑
**10**	*Diplotaxis cretacia*	1	1	1	1	1	1	100	60	40↓	80	100	25↑	100	25↑	100	25↑
**11**	*Diplotaxis erucoides*	1	1	1	1	1	1	0	0	-	80	80	0	100	25↑	100	25↑
**12**	*Diplotaxis gomez-campoi*	1	1	1	1	1	1	90	85	5.56↓	60	90	50↑	100	66.66↑	100	66.66↑
**13**	*Diplotaxis muralis*	1	1	1	1	1	1	100	60	40↓	85	95	11.76↑	50	41.17↓	50	41.17↓
**14**	*Diplotaxis tenuisilique*	1	1	1	1	1	1	100	60	40↓	100	95	5↓	100	0	100	0
**15**	*Diplotaxis viminea*	1	1	1	1	1	1	95	75	21.05	90	85	5.55↓	80	11.11↓	90	0
**16**	*Lepidium sativum*	1	1	1	1	1	1	80	100	25.00↑	85	100	17.64↑	100	17.64↑	100	17.64↑
**17**	*Sinapis alba*	1	1	1	1	1	1	80	80	0	95	85	10.52↓	80	15.78↓	95	0
**18**	*Crambe abyssinica* (EC400058)	3	3	3	3	3	3	90	60	33.33↓	60	65	8.33↑	75	25↑	95	58.33↑
**19**	*Crambe abyssinica* (EC694145)	1	1	1	1	1	1	75	45	40↓	80	80	0	90	12.5↑	90	12.5↑

**20**	*Eruca sativa* (IC57706)	1	1	1	1	1	1	85	75	11.76↓	80	95	18.75↑	95	18.75↑	100	25↑
**21**	*Eruca sativa* (IC62713)	1	1	1	1	1	1	70	60	14.29↓	65	90	38.46↑	85	30.76↑	100	53.84↑
**22**	*Biscutella didyma*	1	3	3	3	3	3	95	40	57.89↓	90	100	11.11↑	100	11.11↓	100	11.11↑
**23**	*Oxycamp*	1	3	3	3	3	3	0	0	-	40	40	0	50	25↑	50	25↑
**24**	*Brassica tournefortii (Rawa)*	1	1	1	1	1	1	65	90	38.46↑	90	80	11.11↓	50	44.44↓	75	16.66↓
**25**	*Brassica rapa* (IC374272)	1	1	1	1	1	1	100	100	0	100	100	0	100	0	100	0
**26**	*B. juncea* (Pusa Jaikisan)	1	1	1	1	1	1	55	50	9.09↓	40	65	62.5↑	60	50↑	90	125↑
**27**	*B. nigra* (EC472708)	1	1	1	1	1	1	100	75	25↓	100	100	0	100	0	100	0
**28**	*B. napus* (GSC 6)	1	1	1	1	1	1	95	90	5.26↓	95	100	5.26↑	100	5.26↑	100	5.26↑
**29**	*B. carinata* (PC-6)	1	1	1	1	1	1	100	80	20↓	100	100	0	100	0	100	0

C: Control and T: Treatment; ↑ and ↓denotes increase and decrease, respectively in germination under stress condition as compared to control

Table S2- Tolerance index (TI) and membership function value (MFV) for trait germination percentage, shoot length and root length under heat stress.

S. no.	Genotypes	Germination Percentage				Root Length				Shoot Length				
		Control	Heat	TI	MFV	Control	Heat	TI	MFV	Control	Heat	TI	MFV	Mean MFV
1	*Brassica fruticulosa*	90	30	0.33	-0.24	2.4	0.9	0.4	0.2	3.3	0.2	0.1	0.8	0.3
2	*Brassica fruticulosa (Spain)*	65	30	0.46	-0.11	1.5	1.3	0.9	0.7	2.5	1.4	0.6	0.5	0.4
3	*Brassica tournefortii (RBT 2002)*	85	75	0.88	0.31	2.1	1.7	0.8	0.6	2.9	2.7	0.9	0.8	0.6
4	*Brassica tournefortii (RBT 2003)*	85	80	0.94	0.37	3.7	0.6	0.2	0.0	3.0	0.7	0.2	0.1	0.2
5	*Camelina sativa*	90	90	1.00	0.43	5.0	0.8	0.2	0.0	2.8	0.3	0.1	0.0	0.1
6	*Capsella-bursapastoris (early)*	95	35	0.37	-0.20	0.8	1.0	1.3	1.1	1.0	0.6	0.6	0.5	0.5
7	*Capsella-bursapastoris (late)*	85	80	0.94	0.37	0.4	0.9	2.1	1.9	1.0	0.7	0.6	0.5	0.9
8	*Diplotaxis assurgens*	80	90	1.13	0.56	1.6	2.4	1.5	1.4	1.7	1.6	1.0	0.9	0.9
9	*Diplotaxis catholica*	95	90	0.95	0.38	2.9	1.6	0.6	0.4	2.3	2.2	1.0	0.9	0.6
10	*Diplotaxis cretacia*	100	60	0.60	0.03	2.7	1.8	0.7	0.5	2.4	2.5	1.0	0.9	0.5
11	*Diplotaxis gomez-campoi*	90	85	0.94	0.38	1.7	2.0	1.2	1.0	2.0	3.3	1.6	1.5	1.0
12	*Diplotaxis muralis*	100	60	0.60	0.03	1.3	1.7	1.3	1.2	1.8	2.4	1.3	1.2	0.8
13	*Diplotaxis tenuisilique*	100	60	0.60	0.03	1.3	1.5	1.2	1.0	1.9	1.6	0.8	0.7	0.6
14	*Diplotaxis viminea*	95	75	0.79	0.22	2.2	1.7	0.7	0.6	2.1	2.4	1.1	1.0	0.6
15	*Lepidium sativum*	80	100	1.25	0.68	4.7	2.3	0.5	0.3	3.1	1.4	0.5	0.4	0.5
16	*Sinapis alba*	80	80	1.00	0.43	7.2	1.0	0.1	0.0	4.2	0.4	0.1	0.0	0.1
17	*Crambe abyssinica(EC400058)*	90	60	0.67	0.10	2.8	2.3	0.8	0.7	3.8	1.3	0.3	0.2	0.3
18	*Crambe abyssinica (EC694145)*	75	45	0.60	0.03	3.1	0.7	0.2	0.1	3.9	1.7	0.4	0.3	0.1
19	*Eruca sativa (IC57706)*	85	75	0.88	0.31	5.5	2.1	0.4	0.2	3.9	2.7	0.7	0.6	0.4
20	*Eruca sativa (IC62713)*	70	60	0.86	0.29	4.7	3.4	0.7	0.6	4.8	0.6	0.1	0.0	0.3
21	*Biscutella didyma*	95	40	0.42	-0.15	1.5	1.5	1.0	0.8	2.1	0.8	0.4	0.3	0.3
22	*Brassica tournefortii (Rawa)*	65	90	1.38	0.82	1.4	2.2	1.6	1.4	2.6	2.4	0.9	0.8	1.0
23	*Brassica rapa var. yellow sarson (NRCPB rapa 8)*	100	100	1.00	0.43	4.4	2.4	0.5	0.4	2.9	1.6	0.6	0.5	0.4
24	*B. juncea (Pusa Jaikisan)*	55	50	0.91	0.34	4.8	2.1	0.4	0.3	3.5	2.5	0.7	0.6	0.4
25	*B. nigra (EC472708)*	100	75	0.75	0.18	6.0	1.7	0.3	0.1	2.8	0.7	0.3	0.2	0.2
26	*B.napus (GSC 6)*	95	90	0.95	0.38	4.2	1.7	0.4	0.2	3.2	0.6	0.2	0.1	0.2
27	*B. carinata (PC-6)*	100	80	0.80	0.23	4.8	1.8	0.4	0.2	3.0	0.7	0.2	0.1	0.2
													Avg	0.5

Table S3-Shoot length and Root length of wild and U triangle species of Brassica germinated under different concentrations of PEG6000. Different letters denote a significant difference at *p* <0.05 based on the least significant difference (LSD) test.

S.no.	Treatment	Control		2.5% PEG				5% PEG				10% PEG			
	Genotype	Shoot length	Root length	Shoot length	Fold change in shoot length	Root length	Fold change in root length	Shoot length	Fold change in shoot length	Root length	Fold change in root length	Shoot length	Fold change in shoot length	Root length	Fold change in root length
1	***Brassica fruticulosa***	2.97^{FEDG}	3.07^{HEGKDJFI}	1.4^{NM}	0.47	1.17^{GF}	0.38	1.27^{MN}	0.43	1.2^{M}	0.39	1.43^{NM}	0.48	1.07^{K}	0.35
2	***Brassica fruticulosa* (Spain)**	2.3^{FHG}	1.13^{ML}	2.17^{HJGI}	0.94	0.63^{G}	0.56	2.57^{HFEIG}	1.12	2.23^{MLK}	1.97	3.1^{FGE}	1.35	4.57^{FHIG}	4.03
3	***Brassica tournefortii* (RBT 2002)**	3.03^{FCEDG}	4.3^{EDF}	2.93^{EDF}	0.97	1.43^{EGF}	0.33	2.57^{HFEIG}	0.85	5.07^{HJGIF}	1.18	2.43^{JIGH}	0.8	6.7^{FED}	1.56
4	***Brassica tournefortii* (RBT 2003)**	2.73^{FEHDG}	2.93^{HGKLJFI}	2.83^{EDF}	1.04	3.77^{EDGCF}	1.28	2.83^{HFEDG}	1.04	4.33^{HJGIK}	1.48	2.8^{FIGH}	1.02	6.23^{FEG}	2.13
5	***Camelina sativa***	2.17^{HG}	2.53^{HGKLJFI}	2.07^{HJKI}	0.95	3.3^{EDGF}	1.3	2.4^{HFJIG}	1.11	5.47^{HEGIF}	2.16	2.93^{FGH}	1.35	9.37^{CBD}	3.7
6	***Capsella bursa-pastoris* (early)**	0.77^{I}	0.63^{M}	1.4^{NM}	1.83	0.5^{G}	0.79	1.07^{N}	1.39	0.77^{M}	1.21	1.57^{NML}	2.04	1.47^{JK}	2.32
7	***Capsella bursa-pastoris* (late)**	0.9^{I}	0.37^{M}	1.13^{N}	1.26	0.5^{G}	1.36	1.2^{MN}	1.33	0.73^{M}	2	2.43^{JIGH}	2.7	1.6^{JK}	4.36
8	***Diplotaxis assurgens***	2.1^{HG}	1.13^{ML}	1.97^{LJKI}	0.94	9.07^{A}	8	1.67^{MKJNL}	0.79	2.07^{MLK}	1.82	2.07^{JMKL}	0.98	2.97^{JKHI}	2.62
9	***Diplotaxis catholica***	2.47^{FEHG}	2.57^{HGKLJFI}	2.27^{HGI}	0.92	2.17^{EDGF}	0.84	2.1^{HKJI}	0.85	1.67^{MLK}	0.65	2.53^{JIGH}	1.03	2.7^{JKI}	1.05
10	***Diplotaxis cretacia***	2.47^{FEHG}	1.83^{HMKLJI}	1.6^{LNKM}	0.65	1.57^{EGF}	0.85	2.07^{HKIJIL}	0.84	2.7^{MJLIK}	1.47	2.23^{JIKL}	0.91	2.47^{JKI}	1.35
11	***Diplotaxis erucoides***	2.2^{FHG}	3.1^{HEGKDJFI}	1.53^{LNM}	0.7	2.37^{EDGF}	0.76	2.13^{HKIJ}	0.97	2.33^{MJLK}	0.75	2.47^{JIGH}	1.12	3.43^{JKHI}	1.11
12	***Diplotaxis gomez-campoi***	2.1^{HG}	1.13^{ML}	1.93^{LJKI}	0.92	1.77^{EGF}	1.56	2^{MKJIL}	0.95	1.47^{ML}	1.29	1.7^{NMKL}	0.81	2.37^{JKI}	2.09
13	***Diplotaxis muralis***	1.8^{IH}	1.43^{MKLJ}	1.3^{NM}	0.72	1.57^{EGF}	1.09	1.7^{MKJNL}	0.94	1.53^{ML}	1.07	1.37^{N}	0.76	2.37^{JKI}	1.65
14	***Diplotaxis tenuisilique***	2.17^{HG}	2.07^{HMGKLJI}	1.63^{LKM}	0.75	1.73^{EGF}	0.84	2.13^{HKIJ}	0.98	1.8^{MLK}	0.87	2.27^{JIKH}	1.05	3.43^{JKHI}	1.66
15	***Diplotaxis viminea***	2.47^{FEHG}	1.23^{MKL}	2.3^{HGI}	0.93	2.17^{EDGF}	1.76	2^{MKJIL}	0.81	3^{MJLIK}	2.43	2.03^{NJMKL}	0.82	2.9^{JKHI}	2.35
16	***Lepidium sativum***	2.87^{FEHDG}	3.83^{EGDF}	3.07^{ED}	1.07	6.03^{BDAC}	1.57	3.2^{FEDC}	1.12	6.17^{HEGDF}	1.61	3.3^{FDE}	1.15	5.4^{FHEG}	1.41
17	***Sinapis alba***	3.2^{FCEBDG}	3.43^{HEGDFI}	2.47^{HGF}	0.77	4.37^{EBDGCF}	1.27	3.63^{BDC}	1.14	8.73^{BDC}	2.54	3.93^{CD}	1.23	11.6^{B}	3.38
18	***Crambe abyssinica* (EC400058)**	3.57^{CEBD}	3.27^{HEGDJFI}	2.8^{EDF}	0.79	3.33^{EDGF}	1.02	4.03^{BA}	1.13	7.2^{EDF}	2.2	3.47^{FDE}	0.97	4.33^{FHIG}	1.33
19	***Crambe abyssinica* (EC694145)**	3.87^{CBD}	3.03^{HEGKJFI}	3.57^{CB}	0.92	3.1^{EDGF}	1.02	4^{BAC}	1.03	6.47^{HEGDF}	2.13	3.43^{FDE}	0.89	4.73^{FHIG}	1.56
20	***Eruca sativa* (IC57706)**	4.33^{B}	4.83^{ED}	3.73^{CB}	0.86	6.17^{BDAC}	1.28	3.33^{BEDC}	0.77	7.1^{EGDF}	1.47	5.47^{A}	1.26	9.43C^{B}	1.95
21	***Eruca sativa* (IC62713)**	5.63^{A}	11.5^{A}	4.9^{A}	0.87	4.47^{EBDGCF}	0.39	4.8^{A}	0.85	10.7^{BAC}	0.93	5.53^{A}	0.98	10.47^{B}	0.91

22	***Biscutella didyma***	2.47^{FEHG}	1.63^{MKLJI}	2.27^{HGI}	0.92	1.73^{EGF}	1.06	2.3^{HKJIG}	0.93	2.4^{MJLK}	1.47	$1.6N^{MKL}$	0.65	2.73^{JKHI}	1.67
23	***Oxycamp***	0	1.3	1.7^{LJKM}	0	1.43^{EGF}	1.1	1.53^{MKNL}	0	1.2^{M}	0.92	2.47^{JIGH}	0	1.4^{K}	1.08
24	***Brassica tournefortii* (Rawa)**	0	2.05	2.6^{EGF}	0	3.1^{EDGF}	1.51	1.67^{MKJNL}	0	2.83^{EDC}	1.38	2.5^{JIGH}	0	4.13^{JFHIG}	2.02
25	***B. rapa* (IC374272)**	3.73^{CBD}	6.97^{D}	3.27^{CD}	0.88	3.83^{EDGF}	0.55	3.17^{FED}	0.85	8.17^{EDC}	1.17	3.33^{FDE}	0.89	7.57^{CED}	1.09
26	***B. juncea* (Pusa Jaikisan)**	3.33^{FCEBD}	3.67^{HEGDF}	3.97^{B}	1.19	5.2^{EBDACF}	1.42	3.33^{BEDC}	1	4.1^{HJLIK}	1.12	3.67^{CDE}	1.1	3.57^{JKHIG}	0.97
27	***B. nigra* (EC472708)**	4.13^{B}	7.83^{C}	3.7^{CB}	0.9	5.43^{EBDAC}	0.69	3.2^{FEDC}	0.77	6.87^{HJLIK}	0.88	4.2^{CB}	1.02	9.67^{CB}	1.23
28	***B. napus* (GSC 6)**	4.2^{B}	10.43^{BA}	3.97^{B}	0.94	7.6^{BAC}	0.73	3.57^{BDC}	0.85	11.2^{BA}	1.07	4.7^{B}	1.12	15.67^{A}	1.5
29	***B. carinata* (PC-6)**	3.23^{FCEBDG}	9.4^{BC}	3.57 CB	1.1	8.43^{BA}	0.9	3.1^{FEDG}	0.96	12.17^{A}	1.29	3.03^{FGE}	0.94	14.6^{A}	1.55

Table S4- Tolerance index (TI) and membership function value (MFV) for trait survival percentage and MDA under heat stress

		Survival Percentage				MDA				
	Genotypes	Control	Heat	TI	MFV	Control	Heat	TI	MFV	Mean MFV
1	*Brassica fruticulosa*	100	27.778	0.278	0.216	0.886022	1.76129	1.987864	1.422075	0.819
2	*Brassica fruticulosa* (Spain)	100	18.605	0.186	0.124	0.16129	0.352688	2.186667	1.620877	0.872
3	*Brassica tournefortii* (RBT 2002)	100	73.913	0.739	0.677	0.301075	1.991398	6.614286	6.048496	3.363
4	*Brassica tournefortii* (RBT 2003)	100	33.333	0.333	0.271	0.550538	0.488172	0.886719	0.320929	0.296
5	*Camelina sativa*	100	29.630	0.296	0.234	0.329032	0.301075	0.915033	0.349243	0.292
6	*Capsella-bursapastoris* (early)	100	75.000	0.750	0.688	0.382796	0.075269	0.196629	0.237581	0.463
7	*Capsella-bursapastoris* (late)	100	85.000	0.850	0.788	0.088172	0.045161	0.512195	-0.07798	0.355
8	*Diplotaxis assurgens*	100	33.333	0.333	0.271	0.094624	0.083871	0.886364	0.320574	0.296
9	*Diplotaxis catholica*	100	36.364	0.364	0.301	0.070968	0.058065	0.818182	0.252392	0.277
10	*Diplotaxis cretacia*	100	38.462	0.385	0.322	0.11828	0.144086	1.218182	0.652392	0.487
11	*Diplotaxis gomez-campoi*	100	39.394	0.394	0.332	0.101075	0.443011	4.382979	3.817189	2.074
12	*Diplotaxis tenuisilique*	100	20.000	0.200	0.138	0.458065	0.843011	1.840376	1.274586	0.706
13	*Diplotaxis viminea*	100	26.923	0.269	0.207	0.791398	0.670968	0.847826	0.282037	0.245
14	*Lepidium sativum*	100	25.000	0.250	0.188	0.051613	0.369892	7.166667	6.600877	3.394
15	*Sinapis alba*	100	39.286	0.393	0.331	0.649462	1.047312	1.612583	1.046793	0.689
16	*Crambe abyssinica* (EC400058)	100	91.176	0.912	0.850	0.247312	0.535484	2.165217	1.599428	1.224
17	*Crambe abyssinica* (EC694145)	100	93.333	0.933	0.871	0.266667	0.735484	2.758065	2.192275	1.532
18	*Eruca sativa* (IC57706)	100	30.769	0.308	0.245	0.574194	1.619355	2.820225	2.254435	1.250
19	*Eruca sativa* (IC62713)	100	16.129	0.161	0.099	0.789247	1.148387	1.455041	0.889251	0.494
20	*Brassica tournefortii* (Rawa)	100	3.125	0.031	-0.031	0.270968	0.535484	1.97619	1.410401	0.690
21	*B. rapa* (NRCPB rapa 8)	100	28.125	0.281	0.219	0.750538	1.019355	1.358166	0.792377	0.506
22	*B. juncea* (Pusa Jaikisan)	100	80.000	0.800	0.738	0.324731	0.690323	2.125828	1.560038	1.149
23	*B. nigra* (EC472708)	100	75.806	0.758	0.696	0.36129	0.713978	1.97619	1.410401	1.053
24	*B.napus* (GSC 6)	100	76.190	0.762	0.700	0.109677	0.84086	7.666667	7.100877	3.900
25	*B. carinata* (PC-6)	100	75.000	0.750	0.688	0.090323	0.576344	6.380952	5.815163	3.251
									Avg	1.187

Table S5- Tolerance index (TI) and membership function value (MFV) for trait shoot length and root length at 2.5% PEG6000

S.no.	Genotype	Control		2.5% PEG					
		**Shoot Length**	**Root Length**	**Shoot Length**	TI	MFV	**Root Length**	TI	MFV
1	*Brassica fruticulosa*	3.0	3.1	1.4	0.5	-0.2	1.2	0.4	0.0
2	*Brassica fruticulosa (Spain)*	2.3	1.1	2.2	0.9	0.2	0.6	0.6	0.2
3	*Brassica tournefortii (RBT 2002)*	3.0	4.3	2.9	1.0	0.2	1.4	0.3	0.0
4	*Brassica tournefortii (RBT 2003)*	2.7	2.9	2.8	1.0	0.3	3.8	1.3	0.9
5	*Camelina sativa*	2.2	2.5	2.1	1.0	0.2	3.3	1.3	0.9
6	*Capsella bursa-pastoris (early)*	0.8	0.6	1.4	1.8	1.1	0.5	0.8	0.4
7	*Capsella bursa-pastoris (Late)*	0.9	0.4	1.1	1.3	0.5	0.5	1.4	1.0
8	*Diplotaxis assurgens*	2.1	1.1	2.0	0.9	0.2	9.1	8.0	7.6
9	*Diplotaxis catholica*	2.5	2.6	2.3	0.9	0.2	2.2	0.8	0.5
10	*Diplotaxis cretacia*	2.5	1.8	1.6	0.6	-0.1	1.6	0.9	0.5
11	*Diplotaxis erucoides*	2.2	3.1	1.5	0.7	0.0	2.4	0.8	0.4
12	*Diplotaxis gomez-campoi*	2.1	1.1	1.9	0.9	0.2	1.8	1.6	1.2
13	*Diplotaxis muralis*	1.8	1.4	1.3	0.7	0.0	1.6	1.1	0.7
14	*Diplotaxis tenuisilique*	2.2	2.1	1.6	0.8	0.0	1.7	0.8	0.5
15	*Diplotaxis viminea*	2.5	1.2	2.3	0.9	0.2	2.2	1.8	1.4
16	*Lepidium sativum*	2.9	3.8	3.1	1.1	0.3	6.0	1.6	1.2
17	*Sinapis alba*	3.2	3.4	2.5	0.8	0.0	4.4	1.3	0.9
18	*Crambe abyssinica (EC400058)*	3.6	3.3	2.8	0.8	0.1	3.3	1.0	0.6
19	*Crambe abyssinica (EC694145)*	3.9	3.0	3.6	0.9	0.2	3.1	1.0	0.7
20	*Eruca sativa (IC57706)*	4.3	4.8	3.7	0.9	0.1	6.2	1.3	0.9
21	*Eruca sativa (IC62713)*	5.6	11.5	4.9	0.9	0.1	4.5	0.4	0.0
22	*Biscutella didyma*	2.5	1.6	2.3	0.9	0.2	1.7	1.1	0.7
23	*B. rapa (IC374272)*	3.7	7.0	3.3	0.9	0.1	3.8	0.6	0.2
24	*B. juncea (Pusa Jaikisan)*	3.3	3.7	4.0	1.2	0.5	5.2	1.4	1.0
25	*B. nigra (EC472708)*	4.1	7.8	3.7	0.9	0.2	5.4	0.7	0.3
26	*B.napus (GSC 6)*	4.2	10.4	4.0	0.9	0.2	7.6	0.7	0.4
27	*B. carinata (PC-6)*	3.2	9.4	3.6	1.1	0.4	8.4	0.9	0.5

Table S6- Tolerance index (TI) and membership function value (MFV) for trait shoot length and root length at 5% and 10% PEG6000

		**5% PEG**						**10% PEG**					
	**Genotype**	**Shoot Length**	TI	MFV	**Root Length**	TI	MFV	**Shoot Length**	TI	MFV	**Root Length**	TI	MFV
1	*Brassica fruticulosa*	1.3	0.4	-0.2	1.2	0.4	0.1	1.4	0.5	-0.1	1.1	0.3	0.2
2	*Brassica fruticulosa (Spain)*	2.6	1.1	0.4	2.2	2.0	1.4	3.1	1.3	0.7	4.6	4.0	3.6
3	*Brassica tournefortii (RBT 2002)*	2.6	0.8	0.1	5.1	1.2	0.6	2.4	0.8	0.1	6.7	1.6	1.1
4	*Brassica tournefortii (RBT 2003)*	2.8	1.0	0.3	4.3	1.5	0.9	2.8	1.0	0.4	6.2	2.1	1.7
5	*Camelina sativa*	2.4	1.1	0.4	5.5	2.2	1.6	2.9	1.4	0.7	9.4	3.7	3.3
6	*Capsella bursa-pastoris (early)*	1.1	1.4	0.7	0.8	1.2	0.7	1.6	2.0	1.4	1.5	2.3	1.9
7	*Capsella bursa-pastoris (Late)*	1.2	1.3	0.6	0.7	2.0	1.5	2.4	2.7	2.0	1.6	4.4	3.9
8	*Diplotaxis assurgens*	1.7	0.8	0.1	2.1	1.8	1.3	2.1	1.0	0.3	3.0	2.6	2.2
9	*Diplotaxis catholica*	2.1	0.9	0.1	1.7	0.6	0.1	2.5	1.0	0.4	2.7	1.1	0.6
10	*Diplotaxis cretacia*	2.1	0.8	0.1	2.7	1.5	0.9	2.2	0.9	0.2	2.5	1.3	0.9
11	*Diplotaxis erucoides*	2.1	1.0	0.2	2.3	0.8	0.2	2.5	1.1	0.5	3.4	1.1	0.7
12	*Diplotaxis gomez-campoi*	2.0	1.0	0.2	1.5	1.3	0.8	1.7	0.8	0.2	2.4	2.1	1.7
13	*Diplotaxis muralis*	1.7	0.9	0.2	1.5	1.1	0.5	1.4	0.8	0.1	2.4	1.7	1.2
14	*Diplotaxis tenuisilique*	2.1	1.0	0.2	1.8	0.9	0.3	2.3	1.0	0.4	3.4	1.7	1.2
15	*Diplotaxis viminea*	2.0	0.8	0.1	3.0	2.4	1.9	2.0	0.8	0.2	2.9	2.4	1.9
16	*Lepidium sativum*	3.2	1.1	0.4	6.2	1.6	1.1	3.3	1.2	0.5	5.4	1.4	1.0
17	*Sinapis alba*	3.6	1.1	0.4	8.7	2.5	2.0	3.9	1.2	0.6	11.6	3.4	2.9
18	*Crambe abyssinica (EC400058)*	4.0	1.1	0.4	7.2	2.2	1.7	3.5	1.0	0.3	4.3	1.3	0.9
19	*Crambe abyssinica (EC694145)*	4.0	1.0	0.3	6.5	2.1	1.6	3.4	0.9	0.2	4.7	1.6	1.1
20	*Eruca sativa (IC57706)*	3.3	0.8	0.0	7.1	1.5	0.9	5.5	1.3	0.6	9.4	2.0	1.5
21	*Eruca sativa (IC62713)*	4.8	0.9	0.1	10.7	0.9	0.4	5.5	1.0	0.3	10.5	0.9	0.5
22	*Biscutella didyma*	2.3	0.9	0.2	2.4	1.5	0.9	1.6	0.6	0.0	2.7	1.7	1.2
23	*B. rapa (IC374272)*	3.2	0.8	0.1	8.2	1.2	0.6	3.3	0.9	0.2	7.6	1.1	0.7
24	*B. juncea (Pusa Jaikisan)*	3.3	1.0	0.3	4.1	1.1	0.6	3.7	1.1	0.4	3.6	1.0	0.5
25	*B. nigra (EC472708)*	3.2	0.8	0.0	6.9	0.9	0.3	4.2	1.0	0.4	9.7	1.2	0.8
26	*B.napus (GSC 6)*	3.6	0.8	0.1	11.2	1.1	0.5	4.7	1.1	0.5	15.7	1.5	1.1
27	*B. carinata (PC-6)*	3.1	1.0	0.2	12.2	1.3	0.8	3.0	0.9	0.3	14.6	1.6	1.1

Table S7-Tolerance index (TI) and membership function value (MFV) for trait germination percentage at 2.5%, 5% and 10% PEG6000

		Germination percentage										Mean
S.no.	Genotype	Control	2.5% PEG	TI	MFV	5% PEG	TI	MFV	10% PEG	TI	MFV	MFV
1	*Brassica fruticulosa*	100.0	45.0	0.5	-0.2	60.0	0.6	-0.5	75.0	0.8	-0.6	-0.2
2	*Brassica fruticulosa (Spain)*	80.0	100.0	1.3	0.5	100.0	1.3	0.4	50.0	0.6	-0.5	0.8
3	*Brassica tournefortii (RBT 2002)*	90.0	95.0	1.1	0.3	95.0	1.1	0.2	100.0	1.1	0.2	0.3
4	*Brassica tournefortii (RBT 2003)*	90.0	90.0	1.0	0.2	100.0	1.1	0.3	100.0	1.1	0.2	0.6
5	*Camelina sativa*	100.0	100.0	1.0	0.2	100.0	1.0	0.2	100.0	1.0	0.1	0.8
6	*Capsella bursa-pastoris (early)*	100.0	100.0	1.0	0.2	100.0	1.0	0.2	100.0	1.0	0.1	0.7
7	*Capsella bursa-pastoris (Late)*	100.0	100.0	1.0	0.2	100.0	1.0	0.2	100.0	1.0	0.1	1.1
8	*Diplotaxis assurgens*	90.0	95.0	1.1	0.3	100.0	1.1	0.3	100.0	1.1	0.2	1.4
9	*Diplotaxis catholica*	80.0	95.0	1.2	0.4	95.0	1.2	0.4	100.0	1.3	0.4	0.3
10	*Diplotaxis cretacia*	80.0	100.0	1.3	0.5	100.0	1.3	0.4	100.0	1.3	0.4	0.4
11	*Diplotaxis erucoides*	80.0	80.0	1.0	0.2	100.0	1.3	0.4	100.0	1.3	0.4	0.3
12	*Diplotaxis gomez-campoi*	60.0	90.0	1.5	0.7	100.0	1.7	0.9	100.0	1.7	0.8	0.7
13	*Diplotaxis muralis*	85.0	95.0	1.1	0.3	50.0	0.6	-0.4	50.0	0.6	-0.3	0.3
14	*Diplotaxis tenuisilique*	100.0	95.0	1.0	0.2	100.0	1.0	0.2	100.0	1.0	0.1	0.4
15	*Diplotaxis viminea*	90.0	85.0	0.9	0.2	80.0	0.9	0.1	90.0	1.0	0.1	0.7
16	*Lepidium sativum*	85.0	100.0	1.2	0.4	100.0	1.2	0.4	100.0	1.2	0.3	0.6
17	*Sinapis alba*	95.0	85.0	0.9	0.1	80.0	0.8	0.0	95.0	1.0	0.1	0.8
18	*Crambe abyssinica (EC400058)*	60.0	65.0	1.1	0.3	75.0	1.3	0.4	95.0	1.6	0.7	0.6
19	*Crambe abyssinica (EC694145)*	80.0	80.0	1.0	0.2	90.0	1.1	0.3	90.0	1.1	0.2	0.5
20	*Eruca sativa (IC57706)*	80.0	95.0	1.2	0.4	95.0	1.2	0.4	100.0	1.3	0.4	0.6
21	*Eruca sativa (IC62713)*	65.0	90.0	1.4	0.6	85.0	1.3	0.5	100.0	1.5	0.6	0.4
22	*Biscutella didyma*	90.0	100.0	1.1	0.3	100.0	1.1	0.3	100.0	1.1	0.2	0.5
23	*B. rapa (IC374272)*	100.0	100.0	1.0	0.2	100.0	1.0	0.2	100.0	1.0	0.1	0.3
24	*B. juncea (Pusa Jaikisan)*	40.0	65.0	1.6	0.8	60.0	1.5	0.7	90.0	2.3	1.4	0.7
25	*B. nigra (EC472708)*	100.0	100.0	1.0	0.2	100.0	1.0	0.2	100.0	1.0	0.1	0.3
26	*B.napus (GSC 6)*	95.0	100.0	1.1	0.3	100.0	1.1	0.2	100.0	1.1	0.2	0.4
27	*B. carinata (PC-6)*	100.0	100.0	1.0	0.2	100.0	1.0	0.2	100.0	1.0	0.1	0.4
											Avg	0.5

Table S8-Tolerance index (TI) and membership function value (MFV) for trait survival percentage at 2.5%, 5% and 10% PEG6000

S. No.	Genotypes	C2.5% PEGSP	T2.5%PEG SP	TI	MFV	C5%PEG SP	T5%PEG SP	TI	MFV	C10%PEG SP	T10%PEG SP	TI	MFV
1	*Brassica fruticulosa*	100.0	100.0	1.0	-0.4	89.7	85.0	0.9	0.9	88.9	79.2	0.9	0.9
2	*Brassica fruticulosa (Spain)*	100.0	100.0	1.0	-0.4	87.5	89.5	1.0	1.0	71.4	66.7	0.9	0.9
3	*Brassica tournefortii (RBT 2002)*	100.0	100.0	1.0	-0.4	82.4	70.8	0.9	0.9	54.5	28.6	0.5	0.5
4	*Brassica tournefortii (RBT 2003)*	100.0	69.4	0.7	-0.7	69.2	38.5	0.6	0.6	63.0	42.9	0.7	0.7
5	*Camelina sativa*	100.0	94.3	0.9	-0.4	83.3	72.0	0.9	0.9	44.4	21.4	0.5	0.5
6	*Capsella bursa-pastoris (early)*	100.0	85.7	0.9	-0.5	71.4	0.0	0.0	0.0	60.0	0.0	0.0	0.0
7	*Capsella bursa-pastoris (late)*	100.0	100.0	1.0	-0.4	82.4	62.5	0.8	0.8	54.5	54.5	1.0	1.0
8	*Diplotaxis catholica*	100.0	100.0	1.0	-0.4	63.6	52.4	0.8	0.8	52.4	50.0	1.0	1.0
9	*Diplotaxis cretacia*	100.0	100.0	1.0	-0.4	87.0	82.1	0.9	0.9	53.3	30.8	0.6	0.6
10	*Diplotaxis muralis*	100.0	100.0	1.0	-0.4	75.0	66.7	0.9	0.9	0.0	0.0	0	0
11	*Diplotaxis viminea*	100.0	100.0	1.0	-0.4	62.5	52.9	0.8	0.8	0.0	0.0	0	0
12	*Lepidium sativum*	100.0	100.0	1.0	-0.4	87.5	77.1	0.9	0.9	50.0	0.0	0.0	0.0
13	*Sinapis alba*	100.0	78.1	0.8	-0.6	63.6	22.7	0.4	0.4	64.3	23.5	0.4	0.4
14	*Crambe abyssinica (EC400058)*	100.0	100.0	1.0	-0.4	60.0	50.0	0.8	0.8	61.9	0.0	0.0	0.0
15	*Crambe abyssinica (EC694145)*	100.0	90.0	0.9	-0.5	63.0	50.0	0.8	0.8	55.6	28.6	0.5	0.5
16	*Eruca sativa (IC57706)*	100.0	78.8	0.8	-0.6	57.9	34.8	0.6	0.6	52.9	50.0	0.9	0.9
17	*Eruca sativa (IC62713)*	100.0	82.8	0.8	-0.6	65.2	36.8	0.6	0.6	54.5	0.0	0.0	0.0
18	*Biscutella didyma*	100.0	88.0	0.9	-0.5	58.3	0.0	0.0	0.0	53.3	0.0	0.0	0.0
19	*Brassica rapa (IC374272)*	100.0	100.0	1.0	-0.4	80.0	72.7	0.9	0.9	64.3	0.0	0.0	0.0
20	*B. juncea (Pusa Jaikisan)*	100.0	100.0	1.0	-0.4	58.3	53.9	0.9	0.9	64.3	16.7	0.3	0.3
21	*B. nigra (EC472708)*	100.0	100.0	1.0	-0.4	64.7	41.4	0.6	0.6	54.5	0.0	0.0	0.0
22	*B.napus (GSC 6)*	100.0	100.0	1.0	-0.4	87.0	71.4	0.8	0.8	53.3	0.0	0.0	0.0
23	*B. carinata (PC-6)*	100.0	78.0	0.8	-0.6	68.4	54.6	0.8	0.8	61.5	0.0	0.0	0.0

Table S9-Tolerance index (TI) and membership function value (MFV) for trait proline content at 2.5%, 5% and 10% PEG6000

S. No.	Genotypes	C2.5 %PEG PRO	T2.5% PEG PRO	TI	MFV	C5% PEG PRO	T5% PEG PRO	TI	MFV	C10 % PEG PRO	T10% PEG PRO	TI	MFV	Mean MFV
1	*Brassica fruticulosa*	0.1	0.2	1.8	1.7	0.1	0.6	4.7	3.5	0.1	0.5	7.5	6.6	2.2
2	*Brassica fruticulosa (Spain)*	0.2	0.7	3.3	3.2	0.1	0.1	1.5	0.3	0.1	0.8	10.1	9.2	2.4
3	*Brassica tournefortii (RBT 2002)*	0.5	0.8	1.6	1.4	0.3	0.7	2.3	1.1	0.1	1.1	10.3	9.3	2.1
4	*Brassica tournefortii (RBT 2003)*	0.5	0.6	1.1	1.0	0.1	0.2	1.4	0.3	0.1	1.1	10.8	9.8	1.9
5	*Camelina sativa*	0.2	0.4	2.8	2.7	0.1	0.8	5.9	4.8	0.1	1.3	9.9	9.0	2.9
6	*Capsella bursa-pastoris (early)*	0.1	0.1	1.3	1.2	0.2	0.5	3.4	2.2	0.1	0.2	2.1	1.2	0.7
7	*Capsella bursa-pastoris (late)*	0.1	0.1	1.9	1.8	0.1	0.2	1.7	0.5	0.1	0.5	6.5	5.6	1.6
8	*Diplotaxis catholica*	0.1	0.3	2.2	2.1	0.0	0.2	4.3	3.1	0.1	0.2	2.7	1.7	1.4
9	*Diplotaxis cretacia*	0.1	0.1	1.8	1.7	0.1	0.1	1.5	0.3	0.1	0.2	2.2	1.2	0.7
10	*Diplotaxis muralis*	0.1	0.6	9.4	9.3	0.2	1.6	9.8	8.6	0.1	0.5	4.9	4.0	4.5
11	*Diplotaxis viminea*	0.1	0.1	1.1	1.0	0.1	0.1	1.1	-0.1	0.0	0.1	1.8	0.9	0.5
12	*Lepidium sativum*	0.2	0.4	2.4	2.3	0.3	1.0	3.8	2.7	0.2	0.7	3.9	2.9	1.4
13	*Sinapis alba*	0.6	0.6	1.0	0.9	0.1	0.6	5.1	3.9	0.8	1.0	1.2	0.2	0.9
14	*Crambe abyssinica (EC400058)*	0.3	0.7	2.5	2.4	0.8	1.6	2.1	1.0	0.1	0.7	10.0	9.1	2.1
15	*Crambe abyssinica (EC694145)*	0.2	0.3	1.1	1.0	0.1	1.5	17.8	16.6	0.3	0.4	1.2	0.2	3.1
16	*Eruca sativa (IC57706)*	0.4	0.7	1.8	1.7	0.2	1.1	4.5	3.4	0.2	0.3	1.9	0.9	1.2
17	*Eruca sativa (IC62713)*	0.3	0.5	1.9	1.8	0.1	1.0	7.5	6.3	0.1	0.4	5.5	4.5	2.1
18	*Biscutella didyma*	0.4	0.6	1.3	1.2	0.1	0.1	1.3	0.1	0.1	0.5	5.6	4.7	0.9
19	*Brassica rapa (IC374272)*	0.2	0.8	3.4	3.3	0.2	0.5	2.9	1.7	0.2	0.6	3.0	2.0	1.3
20	*B. juncea (Pusa Jaikisan)*	0.6	0.9	1.5	1.4	0.1	1.6	13.3	12.1	1.2	1.1	0.9	-0.1	2.4
21	*B. nigra (EC472708)*	0.7	1.0	1.5	1.4	0.3	1.6	4.8	3.6	0.5	1.4	3.0	2.0	1.2
22	*B.napus (GSC 6)*	0.6	0.9	1.5	1.3	0.1	1.1	8.0	6.8	0.1	1.0	6.5	5.5	2.4
23	*B. carinata (PC-6)*	0.6	1.0	1.8	1.7	0.3	1.3	4.4	3.2	0.6	1.2	2.0	1.1	1.0
													Avg	1.77
